# Histopathological Features and Composition of Gut Microbiota in Rhesus Monkey of Alcoholic Liver Disease

**DOI:** 10.3389/fmicb.2019.00165

**Published:** 2019-02-08

**Authors:** Hong Wang, Yaping Yan, Xiaoyan Yi, Yanchao Duan, Junfeng Wang, Shanshan Li, Lilin Luo, Tianzhuang Huang, Briauna Inglis, Xi Li, Weizhi Ji, Tao Tan, Wei Si

**Affiliations:** ^1^Yunnan Key Laboratory of Primate Biomedical Research, Institute of Primate Translational Medicine, Kunming University of Science and Technology, Kunming, China; ^2^Shenzhen Institutes of Advanced Technology, Chinese Academy of Sciences, Shenzhen, China; ^3^Department of Hepatic and Bile Duct Surgery, The First People's Hospital of Yunnan Province, Kunming, China; ^4^Department of Pathology, The First People's Hospital of Yunnan Province, Kunming, China

**Keywords:** gut microbiota, histopathological features, alcoholic liver disease, metagenomic sequencing, rhesus monkey

## Abstract

Alcohol-induced chronic liver disease (ALD) is becoming the most common liver disease in the world. However, there are no effective, universally accepted therapies for ALD. The etiology of ALD remains blurry so far. Historical evidence has demonstrated a link between the liver and gut microbiota. But it is difficult to distinguish the effect of gut microbiota changes caused by alcohol consumption in humans since the microbiota change detected in humans is complicated by diet and environmental factors. Due to the genetic, physiological, metabolic, and behavioral similarities to humans, the rhesus monkey provides excellent translational validity in preclinical studies, and the diet and environmental conditions can be controlled well in rhesus monkey. In our study, we explored the relationship between ALD and the gut microbiome in the rhesus monkeys with alcoholic liver steatosis. Our results showed that there was a change of the bacterial community structure in monkeys with ALD. Differences of the relative abundances of gut microbiota at phylum, order, family, genus, and species levels were observed between control monkeys and monkeys with ALD, and different pathways enriched in the monkeys with ALD were identified by metagenomic function analysis. Firmicutes, Proteobacteria, Verrucomicrobia tended to increase whereas Bacteroidetes and Actinobacteria decreased in the fecal microbiota of ALD group compared to the control group. Lactobacillales and *Lactobacillus* significantly decreased in ALD monkeys compared with normal monkeys, *Streptococcus* was lower in the ALD group compared with the control group. The non-human primate model of ALD will be useful for exploration of the microbiome markers as diagnosis and potentially prognosis for ALD. The ALD model will benefit the development of new therapeutic procedures for treating ALD and provide safety and efficacy evaluation for clinical application.

## Introduction

Alcohol-induced chronic liver disease (ALD) has been one of the leading causes of liver cirrhosis and liver cancer related deaths world for decades, also causing severe medical and economic burdens (Gao and Bataller, 2011). However, the etiology of ALD remains blurry. Rapid advances in sequencing technology have enabled to identify about 1,000 bacterial species and 7,000 bacterial strains, and there are probably 10^13^-10^14^ microbiota exist in the intestinal track (Consortium HMP., 2012; Westfall et al., 2017). Previous studies have shown a connection between gut microbiota and the liver (Abushanab and Quigley, 2010). Gut microbiota make up about 70% of the all microbiota community, Firmicutes and Bacteroidetes are the two dominated phyla, where Firmicutes is about 35–80% and Bacteroidetes comprises roughly 17–60% of the gut microbiota (Qin et al., 2010; Consortium HMP., 2012). Actinobacteria, Proteobacteria, Verrucomicrobia have correspondingly low abundance in the intestinal (Palmer et al., 2007). Change in the proportion of Bacteroidetes to Firmicutes in the gut microbiota has been revealed to be related to many diseases, such as irritable bowel syndrome (Carroll et al., 2011), cancer (Schwabe and Jobin, 2013), and obesity (Lelouvier et al., 2016).

There are bidirectional links in the gut and liver through the biliary tract, portal vein, and systemic circulation system was termed as “gut-liver axis” (Quigley, 2017). In the intestine, metabolic products of microorganisms (such as bile acids and amino acids), which translocate to the liver through the portal vein further influence liver functions (Stärkel and Schnabl, 2016). A healthy balanced gastrointestinal (GI) system ensures microorganisms to co-exist with the host, which has many functions, such as maintaining a supply of essential nutrients, immune response, metabolism, and gut structure (Hooper and Gordon, 2001). In a GI disordered state, the abundance of gut bacterial species change, and the less abundance strains start to dominate the microbial community. At same time, gut dysbiosis will lead to increased intestinal permeability, which results in the overgrowth of bacteria, translocation of microorganisms, and microbial products including short chain fatty acids and lipopolysaccharides (Segawa et al., 2008; Hartmann et al., 2015). The intake of alcohol affects the diversity and composition of gut microbiota in the GI system, and these changes in the composition of gut microbiota will have negative on the host. Furthermore, alcohol consumption also contributes to neuroinflammation by gut dysbiosis, subsequently bringing about the mental symptoms of alcoholism (Gorky and Schwaber, 2015). Studies have shown that Bacteroidetes abundance were significantly lower compare with healthy controls in alcoholics, while the abundances of Proteobacteria were significantly higher in healthy control group to alcoholics (Mutlu et al., 2012).

Liver biopsy is a bench mark for characterizing liver histology in liver disease patients. On the contrary, this procedure is invasive and can lead to negative side effects like risk of infection and post-operative pain, and requires prolonged care. Therefore, there is a present need for the development of a novel biomarker-based diagnostic strategy. Microbiome can be used markers to diagnosis or potentially prognosis of disease and predict the outcome of treatment strategy by analysis of patient microbiota. On the other hand, animal models are important for treat ALD by showing ALD pathology. Since the genetic, physiological, metabolic, and behavioral similarities to humans, non-human primates would as good animal models to accurately express the metabolic and histological features of human ALD. In our previous study, we generated a non-human primate model with ALD, which cover the clinical progression of biochemistry, pathology, and hepatic gene expression in humans with ALD (Wang et al., 2015). Now we explored the relationship between alcoholic liver disease and the gut microbiome in the rhesus monkeys with alcoholic liver steatosis in an effort to understand the etiology of ALD with deep insight, and attempted to find difference strains between monkey of ALD and normal monkey, there difference strains can be used as biomarker to diagnosis or auxiliary diagnosis of ALD. Human gut microbiota are easily affected by the environment, diet and drugs, and our study on the rhesus monkeys has well-avoided the interference of these aspects. More importantly our study can more accurately express the intestinal microbial state of liver diseases, providing valuable reference for the microbial therapy of human alcoholic liver in the future.

## Materials and Methods

### Animal

Sixteen adult rhesus monkeys individually caged and randomly assigned to two groups (*n* = 8/group). All of animals were maintained a 12 h light: 12 h darkness cycle, temperature was kept at 18–26°C and humidity from 40 to 70%. All procedures were approved by the Institutional Animal Care and Use Committee of Kunming University of Science and Technology (protocol number: KUST-2016-18), and were carried out in accordance with the Guide for the Care and Use of Laboratory Animals (8th edition). Eight rhesus monkeys (ID: 98386, 98380, 99824, 98335, 99353, 99352, 98333, 25) were given 24 h access to a solution of sugared ethanol for about 3 years. The beginning concentration of ethanol was 5% (v/v), and the ethanol concentration increased by 5% per week until it finally reaches a 25% concentration (Wang et al., 2015). The other eight control animals (ID: 3017, 30028, 4054, 7421, 4016, 3085, 30052, 2368) given free access to water instead of alcohol were used as control group.

### Serum Biochemical Measures

Rhesus monkeys were anesthetized by ketamine chloride (10 mg/kg; Shenyang Veterinary Pharmaceutical Inc., China), blood samples were obtained from the femoral vein after fasting for 12 h. Plasma chemistry were measured by Roche Modular P800 automatic biochemical analyzer (Roche Diagnostics Ltd., Basel, Switzerland): aspartate aminotransferase (AST, U/l), alanine aminotransferase (ALT, U/l), γ-glutamyl transpeptidase (GGT, U/l), lactate dehydrogenase (LDH, U/l), alkaline phosphatase (ALP, U/l), globulin (GLOB, g/L), albumin (ALB, g/L), total protein (TP, g/L).

### Histological Analysis

Before the liver biopsy procedure, all of the monkeys were anesthetized with ketamine chloride (10 mg/kg) and morphine hydrochloride (0.2 mg/kg, Northeast Pharmaceutical Group, China) by intramuscular injection. With the assistance of guidance of an ultrasound system (Dynamic Imaging Ltd., Livingston, Scotland, UK) equipped with a 5–10 MHz linear-array transducer, liver biopsies were performed using a Bard Monopty biopsy gun (Bard Biopsy Systems, Tempe, AZ, USA) loaded with a 16-gauge and echogenic-coated tip disposable biopsy needles (Bard Peripheral Vascular, Inc. USA) with a single pass by the percutaneous route in the right lower intercostal space as our previous report (Wang et al., 2015). The liver tissue specimen from each monkey was fixed in 10% neutral buffered formalin and embedded in paraffin. Then the paraffin-embedded tissues were sectioned and stained with haematoxylin and eosin (H&E) for subsequent histopathological analysis.

### Sample Collection and DNA Extraction

Fresh fecal samples were collected from control and ALD groups were placed in sterile tubes and transferred to the laboratory immediately in an ice bath after the confirmation of the success of modeling evaluated by serum biochemical measures and histological analysis. All of the fecal samples were stored at −80°C. Isolation of purified microbial genomic DNA was performed from each fecal sample using MoBio PowerSoil® DNA Extraction Kit (arlsbad, CA, USA) according to the manufacturer's recommendation. DNA concentration was measured using Qubit® DNA Assay Kit in Qubit® 2.0 Flurometer (Life Technologies, CA, USA) in 1 month.

### Library Preparation for Sequencing

Each sample need a total amount of 700 ng DNA, these DNA was used as input material for the DNA sample preparations. According to manufacturer's recommendation, sequencing libraries were generated using NEB Next® Ultra DNA Library Prep Kit for Illumina® (NEB, USA), and index codes were added to attribute sequences for each sample.

### Clustering and Sequencing

In the cBot Cluster Generation System, the clustering of the index-coded samples was performed by HiSeq 4,000 PE Cluster Kit (Illumia) according to the manufacturer's instructions. After cluster generation, the library preparations were sequenced on an Illumina Hiseq 4,000 platform and 150 bp paired-end reads were generated.

### Microbial Bioinformatic Analysis

Raw paired-end reads were processed for quality control by removing: (1) reads with adaptors, (2) low-quality reads that have more than 40% of bases with a quality score value <5, (3) reads containing more than 10% unknown bases, and (4) reads that mapped to host genome. Then, SOAP *de novo* (http://soap.genomics.org.cn/soapdenovo.html) was used to assemble the high-quality reads by testing different k-mer. The Scaffolds with the biggest N50 were selected and cut into contigs (termed Scaftigs) at ambiguous Ns and the contigs longer than 500 bp were saved. USEARCH software was then applied to remove redundant Scaftigs, and all high-quality reads were aligned to these non-redundant Scaftigs by SoapAligner. The depth of each Scaftigs was calculated as the sum of all mapped reads.

Furthermore, these Scaftigs were used for Taxonomic annotation by aligning them with Bacteria, Fungi, Archaea, and Viruses sequences from NT (Version: 2014) database of NCBI by BLAST algorithm (evalue ≤ 1e-5). Then, a lowest common ancestor (LCA) classification was performed on DIAMOND results using MEGAN with default parameters. Relative abundance of each taxonomy is generated based on LCA annotation and depth of Scaftigs. Alpha diversity analysis was displayed with R software (v2.15.3). The difference tests of Alpha diversity for different groups were performed using Wilcoxon Rank Sum Test. Beta diversity on unweighted UniFrac were calculated by QIIME software (v1.7.0).

All these contigs were applied for gene prediction using MetaGeneMark (Noguchi et al., 2006) for each sample using a length threshold of 300 bp. Then the non-redundant gene catalog was constructed by pairwise comparison of all the predicted ORFs with CD-HIT (Li and Godzik, 2006) and the redundant genes were removed using a sequence identity cut-off of 0.95. Additionally, the functional annotation of the non-redundant genes were performed at different levels of the Kyoto Encyclopedia of Genes and Genomes (KEGG) database (Ref).

Metastats analysis was conducted to investigate the difference of the relative abundance for each species and gene between the two groups (White et al., 2009). A multi-comparison adjusted *Q* < 0.05 was used to define significant differences.

A metagenomic biomarker discovery approach called Linear discriminant analysis effect size (LEfSe) was used to identify the genomic features which were different between various classes. Kruskal-Wallis and pairwise Wilcoxon tests were implemented, followed by a Linear discriminant analysis (LDA) to evaluate the effect size determined by LEfSe of each differentially abundant taxon. Bacteria with considerably increased values were defined as those with an LDA score (log10) of over 2. Less than 0.01% of the total bacterial reads, corresponding with ≤ 10^7^ CFU/g feces, were omitted from further analysis due to low and inaccurate read counts, although significant LDA scores were observed.

### Statistical Analysis

All of the plasma chemistry parameters were expressed as the mean ± SEM. Plasma chemistry parameters (GGT, AST, ALT, AST/ALT ratio, LDH, ALP, TP, GLOB, ALB, and A/G) were analyzed by Student *t*-test to observe differences between the levels of serum plasma makers of control and experimental groups. A *p* < 0.05 was considered to be significant for all analyses.

## Results

### Plasma Chemistry Features of Alcohol-Fed Rhesus Monkeys

The number of control monkeys and monkeys with alcohol induced liver steatosis were 8 and 8, respectively. Animal information including gender, bodyweight were summarized in Table 1. The liver function and blood serum lipids from each group were summarized in Table 2. Blood plasma GGT concentrations were significantly elevated in monkeys with ALD compared to the control group (*p* < 0.05). AST concentrations of monkey with ALD were increased (*p* < 0.05). The ratio of AST/ALT as independent predictor of ALD was exceeded one, which reflects the presence of ALD in those monkeys. The elevated level of serum plasma LDH of liver steatosis group indicated liver damage in these animals (*p* < 0.05).

**Table 1:** Information about control monkeys and monkeys with alcohol induced liver steatosis.

**Animal**	**Group**	**Gender**	**Bodyweight (Kg)**
98386	ALD	female	9.2
98380	ALD	female	5.8
99824	ALD	female	3.7
99352	ALD	female	6
99353	ALD	male	7.7
99335	ALD	male	12
98333	ALD	male	7.5
25	ALD	male	6.3
2386	Control	female	7.2
30028	Control	female	6.3
4054	Control	female	7.4
30052	Control	female	6.1
4016	Control	female	8.6
3085	Control	male	7.9
7421	Control	male	7.5
3017	Control	male	5.8

**Table 2:** The serum plasma chemistry parameters in control rhesus monkeys and rhesus monkeys with alcohol induced liver steatosis.

**Variables**	**Control (mean ± SE)**	**ALD (mean ± SE)**
GGT (U/L)*	53 ± 5.6	77.4 ± 10.3
AST (U/L)*	24 ± 4.6	41.8 ± 6.8
ALT (U/L)	25.6 ± 3.4	33.6 ± 6.1
AST/ALT*	0.1 ± 0.1	1.3 ± 0.1
LDH (U/L)*	251.6 ± 27.9	371.8 ± 48.8
ALP (U/L)	197.9 ± 29.2	250.9 ± 52.5
TP (g/L)	67.5 ± 4.6	66.6 ± 4.7
GLOB (g/L)	28.8 ± 2.9	29.7 ± 2.2
ALB (g/L)	38.8 ± 3.5	36.9 ± 2.9
A/G	1.4 ± 0.2	1.3 ± 0.1

*Serum plasma chemistry parameter with an asterisk indicates a significant difference (p < 0.05) between control rhesus monkeys and rhesus monkeys with alcohol induced liver steatosis*.

### Histological Features of Alcohol-Fed Rhesus Monkeys

**Figure 1:**
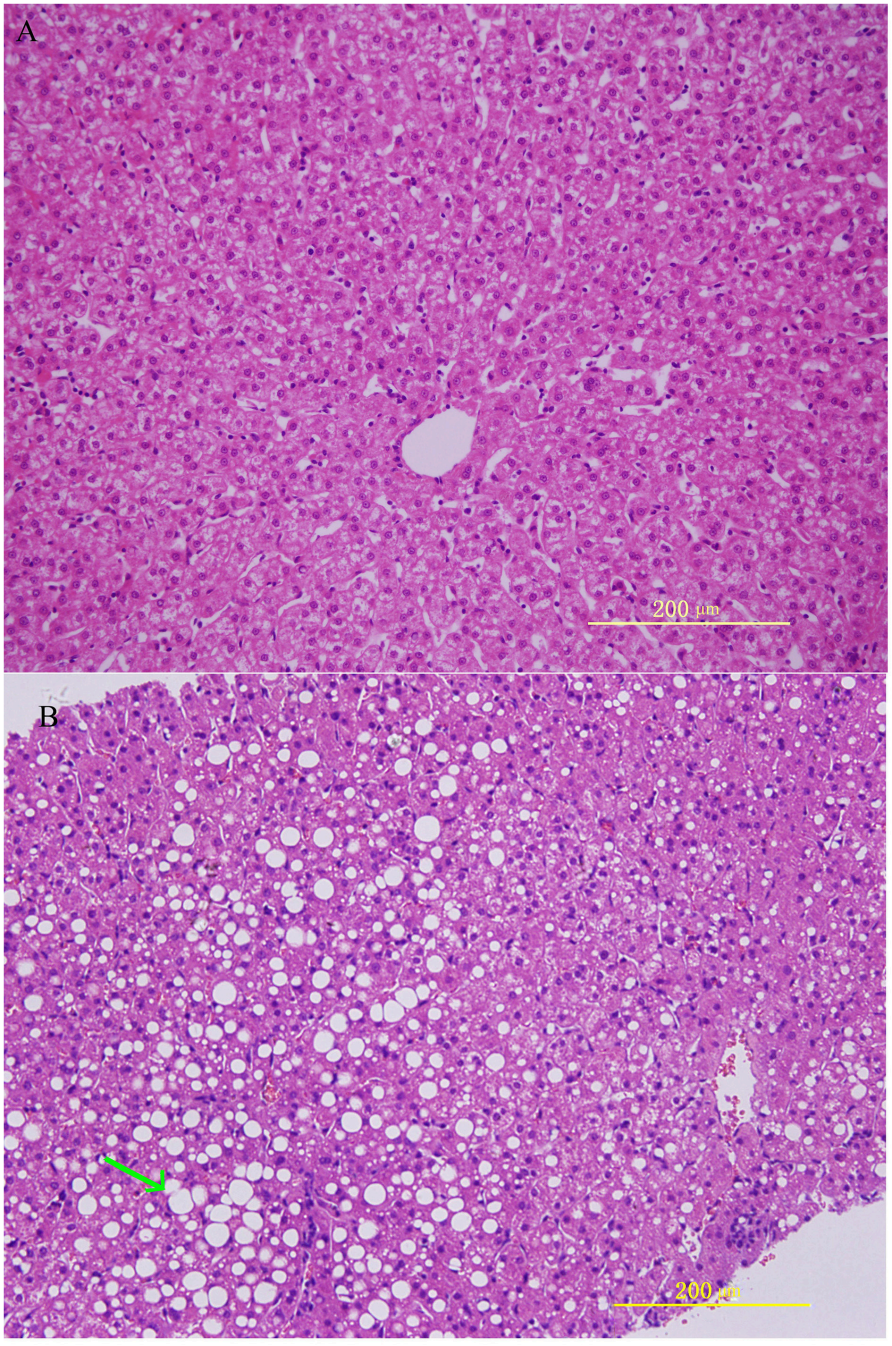
Histological features in rhesus monkeys with control rhesus monkey liver **(A)** and alcohol induced liver steatosis. **(B)** Obvious hepatocellular vacuolization and macro- and micro-vesicular lipid accumulation can be observed in the liver hepatocytes.

HE staining was performed on the liver sections. Rhesus monkeys from the experimental group demonstrated noticeable steatosis, including macro lipid or micro-vesicular lipid accumulation in liver hepatocytes as well as hepatocellular vacuolization. At same time, inflammation and ballooning degeneration were also observed (Figure 1).

### Gut Microbiota Composition Between Control and ALD Monkeys

**Figure 2:**
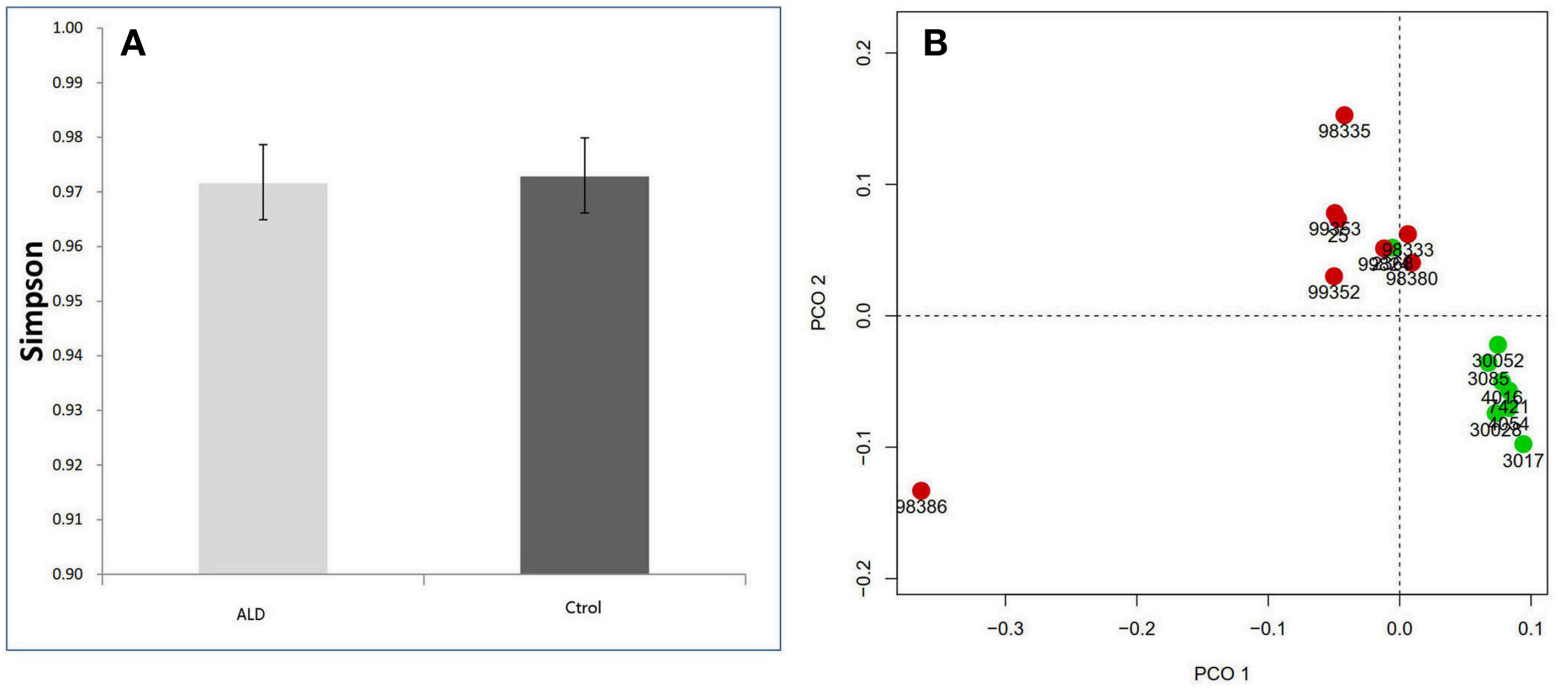
**(A)** Simpson index based on the OTU counts. **(B)** PCA analysis based on unweighted Unifrac distance.

The monkey fecal microbiota was characterized by metagenomics sequencing. Alpha diversity analysis showed that the stool microbiota of ALD monkeys was less diverse compared to controls (Simpson's Index), but no statistical difference was observed (Figure 2A). By examining the unweighted UniFrac distance, ALD monkeys, and healthy controls were separated on the PCoA plot (Figure 2B).

**Figure 3:**
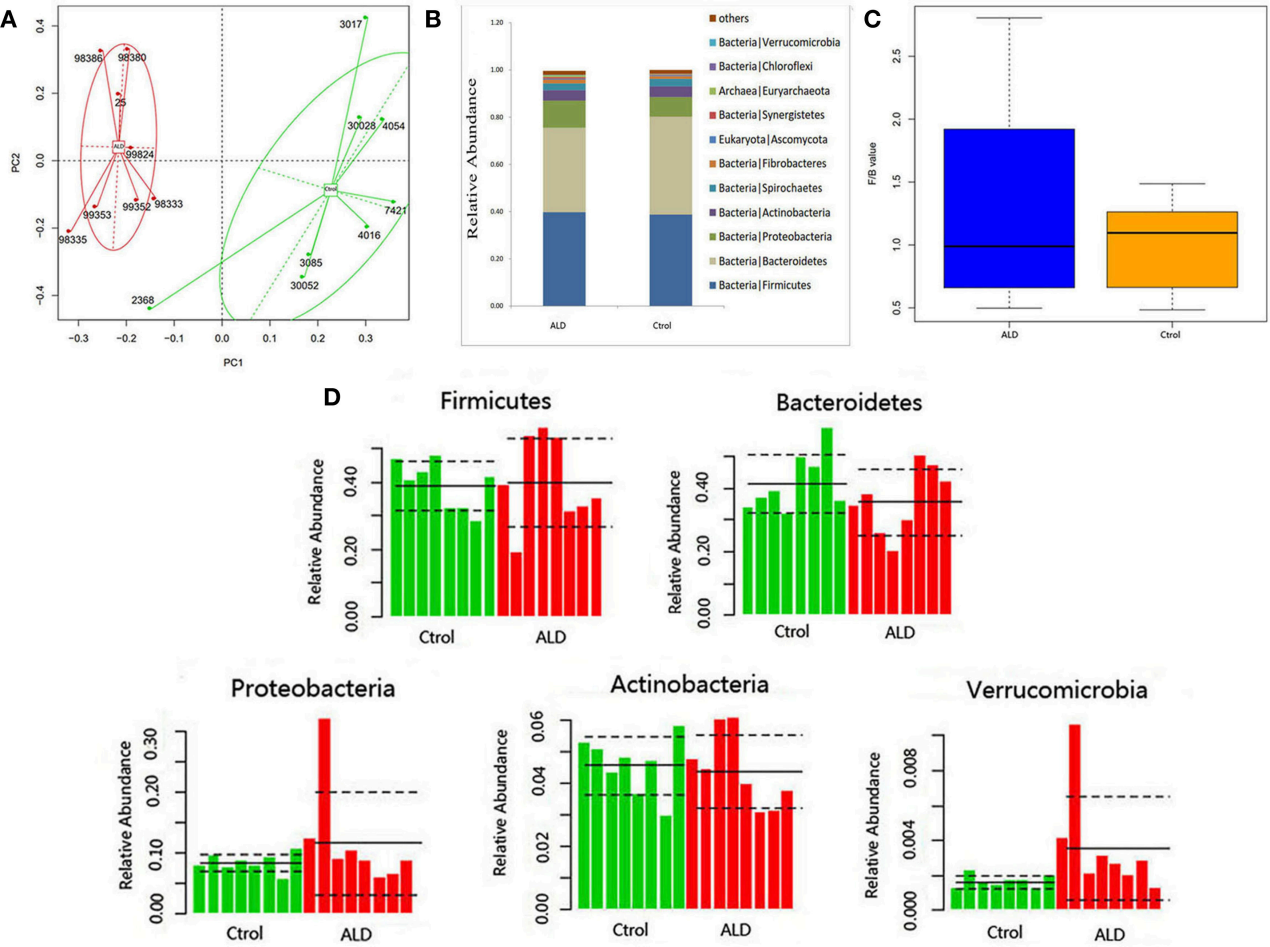
**(A)** A PCoA analysis between ALD group and control group. **(B)** Relative abundance at the phyla. **(C)**The value of Firmicutes vs. Bacteroidetes of control and ALD monkeys. **(D)** The relative abundance of five main phyla of control and ALD monkeys.

With regards to the analysis of bacteria differential abundance. A PCoA analysis on the relative abundance indicated a considerable separation between these two groups, suggesting a change of bacterial community structure in monkeys with ALD (Figure 3A). The gut microbiota in the control group and ALD group are both dominated by four phyla: Bacteroidetes, Firmicutes, Protebacteria, Actinobacteria at the phylum level (Figure 3B). The value of Firmicutes vs. Bacteroidetes (F/B value) decreased in the ALD group (Figure 3C). The median relative abundance decreased in Bacteroidetes and increased in Proteobacteria in the ALD group compared to the control group. Interestingly, a high level of Verrucomicrobia was found in the ALD group (*p* = 0.0209; Figure 3D).

At the order level (Figure S1), higher abundance of Cytophagales (*p* = 0.046), Flavobacteriales (*p* = 0.0011), Sphingobacteriales (*p* < 0.0001), Lactobacillales (*p* = 0.0117), Nitrosomonadales (*p* = 0.0274), Opitutales (*p* = 0.0274), Helotiales (*p* = 0.0274), Ophiostomatales (*p* = 0.0357) were observed in the population of healthy controls compared to ALD monkeys.

**Figure 4:**
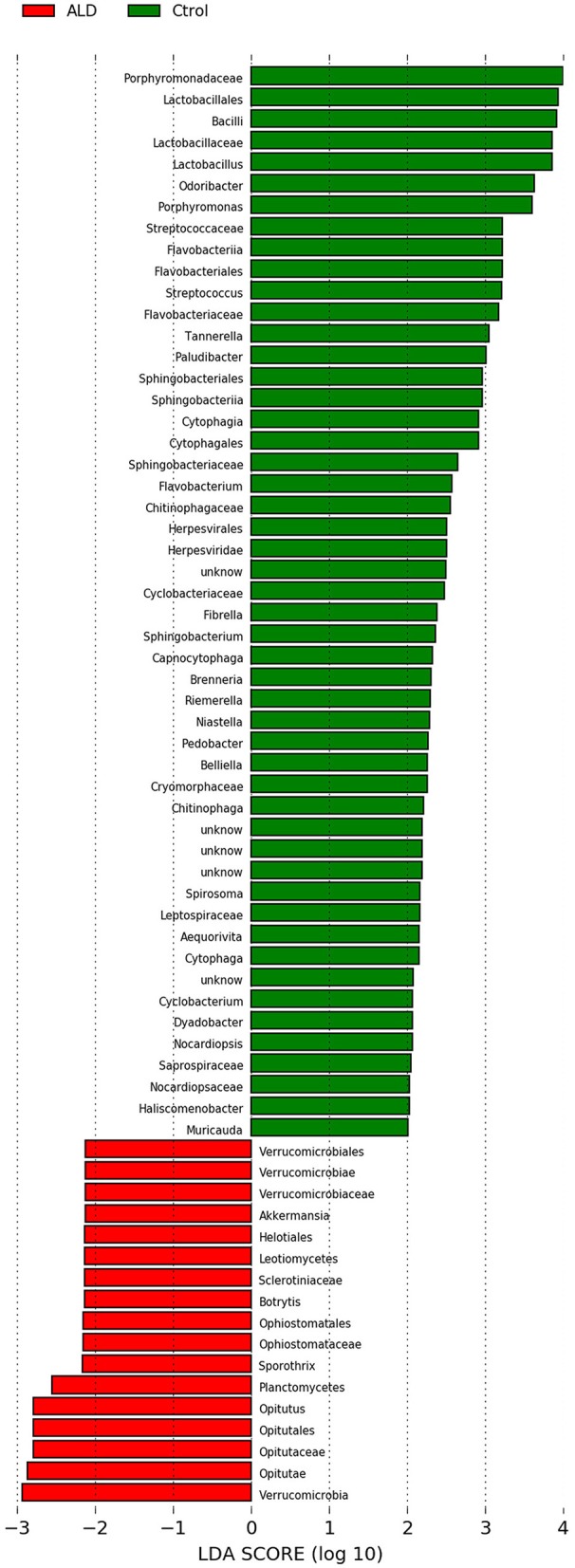
Linear discriminant analysis (LDA) effect size (LEfSe) analysis revealed significant bacterial differences in fecal microbiota between the ALD (positive score) and control groups (negative score).

**Figure 5:**
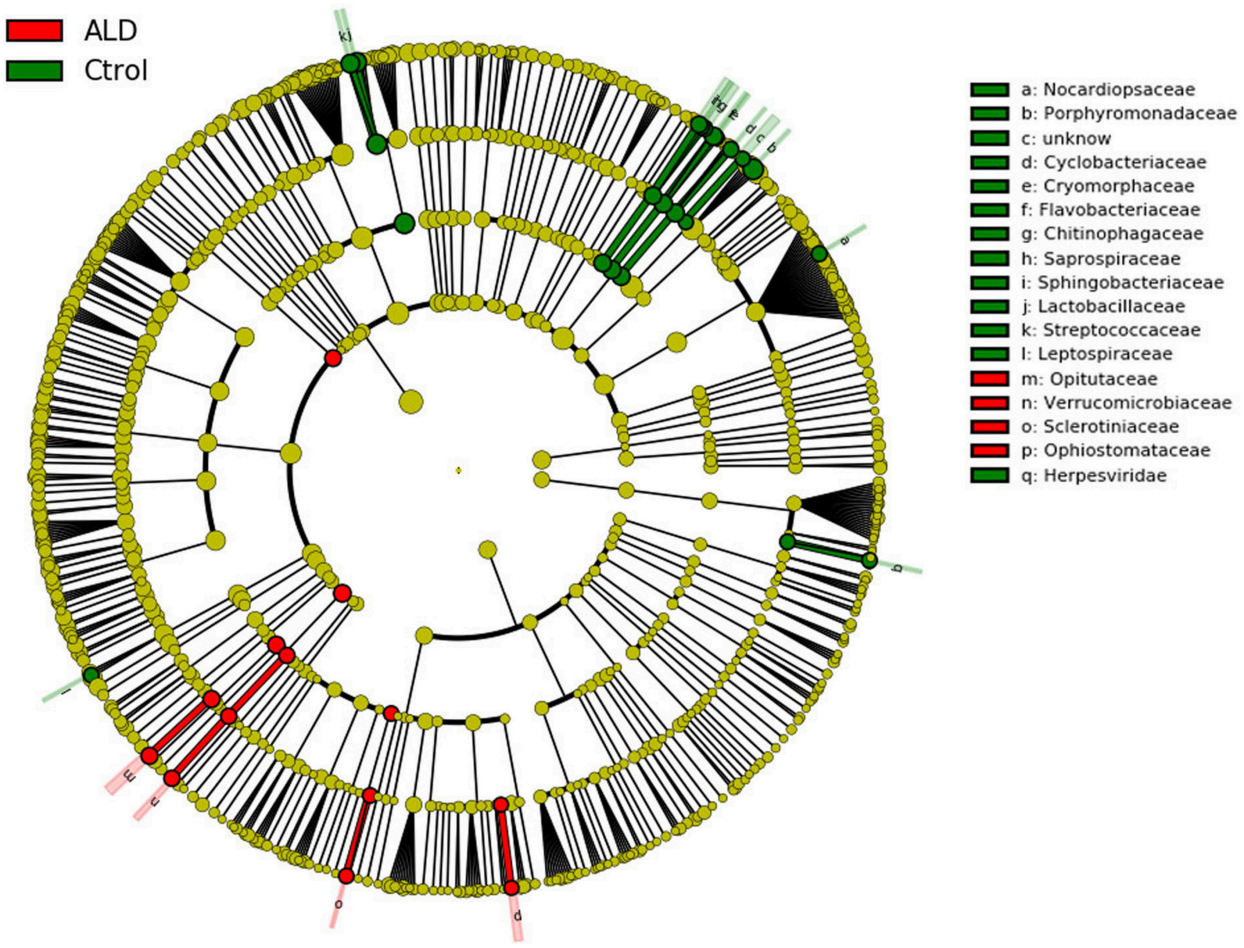
Cladogram using LEfSe method indicating the phylogenetic distribution of fecal microbiota associated with ALD and control subjects.

At the genus level, significant differences between ALD group and control group were observed in 30 genera (Figure S2). According to linear discriminant analysis LEfSe analysis, our results reflected a significant difference in gut microbiota between the ALD and control groups. Here, we particularly considered differences at the genus level of gut microbiota. The relative abundances of *Optitutus* (*p* = 0.0274), *Botrytis* (*p* = 0.0274), *Sporothrix* (*p* = 0.0357) were higher in the ALD group than in the control group at the genus level, whereas there are 22 genera that were more dominant in the control group, such as *Lactobacillus* (*p* = 0.0157), *Streptococcus* (*p* = 0.0274), *Brenneria* (*p* = 0.0082), *Tannerella* (*p* = 0.0117), and so on (Figures 4, 5).

At the species level, 50 species showed significant differences between the ALD group and the control group (Figure S3), and 44 species showed a higher prevalence in the control group. ALD monkeys showed higher abundance of *Clostridium cellulolyticum* (*p* = 0.0357), *Acidaminococcus fermentans* (*p* = 0.046), *Escherichia coli* (*p* = 0.046), *Opitutus terrae* (*p* = 0.0357), *Botrytis cinerea* (*p* = 0.0274), *and Sporothrix schenckii* (*p* = 0.0357).

### Predictive Function Analysis

**Figure 6:**
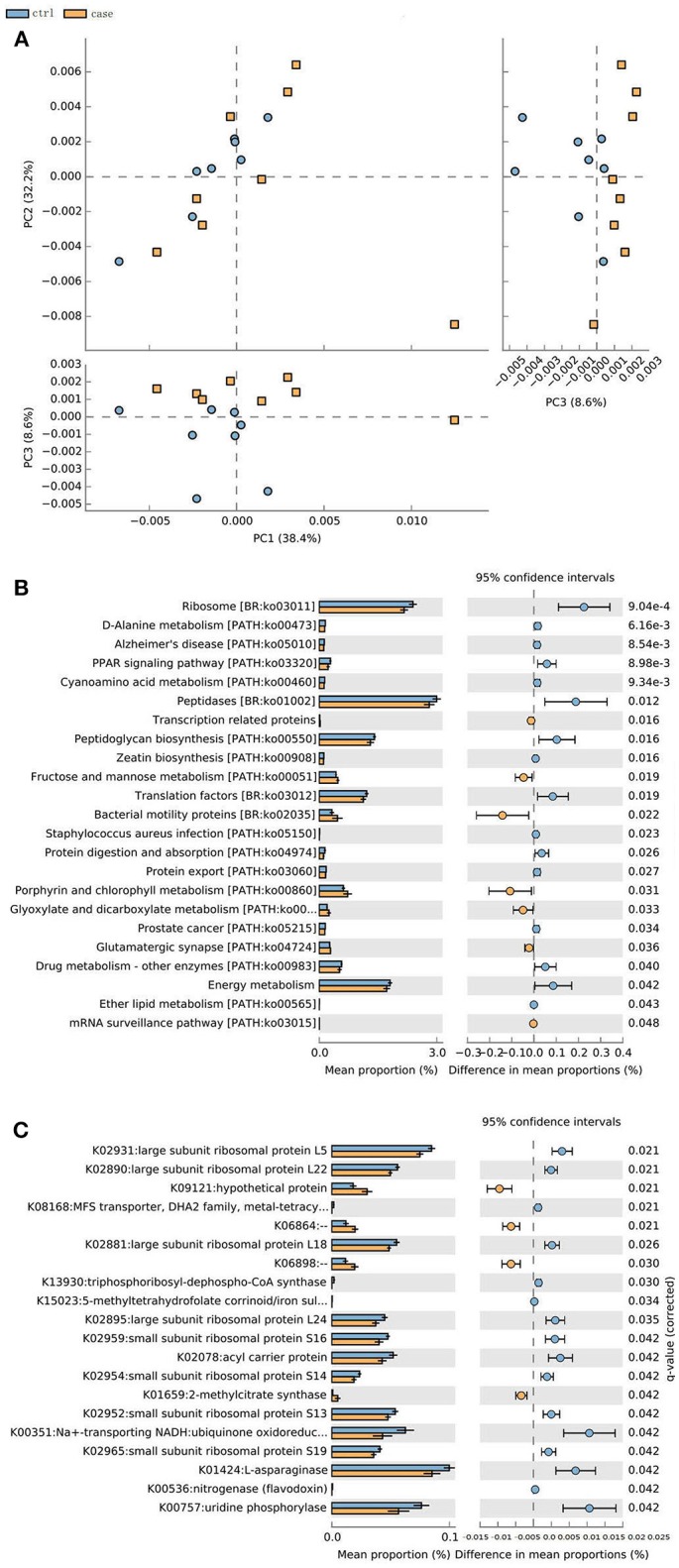
**(A)** PCoA analysis of all the samples based on gene profiles. The different colors designate samples from different groups, The PCoA demonstrates a separation at the gene level. **(B)** 20 KOs were significantly different in the ALD and control group. **(C)** 23 significantly different pathways were enriched in the ALD and control group.

The metagenome data allowed us to identify an updated gene catalog that contains 1,713,579 non-redundant genes in total, with an average of 258,256 non-redundant genes for each sample. This gene catalog can be classified into 4,788 KOs and 249 pathways. A PCA analysis on the relative abundance of the KO profile showed a considerable separation between the ALD groups and control group (Figure 6A). Based on these KOs, we identified that 20 KOs were significantly different in the ALD and control group (Figure 6B). Furthermore, 23 significantly different pathways were enriched in the ALD and control groups (Figure 6C).

## Discussion

Non-human primates are very suitable and useful animal models for studying the pathogenesis of ALD. In our study, we generated an ALD rhesus model by feeding them a 25% ethanol solution for about 3 years, with 8 of the rhesus monkeys showing steatosis by histological evaluation and serological test, and we demonstrated that alcohol intake had a significant impact on rhesus monkey gut microbial diversity. The fecal microbiota of ALD monkeys was less diverse compared to controls but with no significant differences observed, which may be due to the fact that a limited number of monkeys were involved in this study since ALD monkeys are valuable and rare. However, the unweighted UniFrac distance analysis indicated a clear difference in the gut microbial composition of ALD monkeys compared to controls. In the present study, the monkey (98386) was separated from the ALD groups from the PCoA analysis, and the abundance of Escherichia and Helicobacter in this monkey significantly increased compared to other ALD monkeys, though the histological examination of this monkey also showed obvious steatosis. In a previous study, the abundance of Escherichia and Helicobacter was significantly increased in diarrhea predominant irritable bowel syndrome and ulcerative colitis compared to healthy man (Zhong et al., 2017). According to the veterinarian care record at the point of sampling, this monkey had unknown cause of mild intermittent diarrhea, which might be the reason of the separation in the PCoA analysis. These findings suggest that alcohol intake is the pivotal factor affecting the entire composition of the gut microbiota.

In the present study, PCoA analysis at the phyla level demonstrated a clear separation of gut microbiota between control and ALD monkeys. Bacteroidetes, Firmicutes, Proteobacteria, and Actinobacteria dominated the fecal microbial communities in ALD and control groups, a lower abundance of Bacteroidetes and a higher abundance of Proteobacteria were exhibited in the ALD monkeys compared with those of normal rhesus monkeys. Our results are consistent with the findings in humans that ALD patients also exhibited a dysbiosis with lower abundances of Bacteroidetes and higher abundances of Proteobacteria (Keshavarzian et al., 2001). A previous study also has shown that overpropagation of gram-negative bacteria from the Proteobacteria phylum due to chronic ethanol consumption leads to increase plasma endotoxin levels and hepatic inflammation (Bullotterson et al., 2013). The increase of the Firmicutes/Bacteroidetes ratio has been suggested as one of the hallmarks of human gut microbiota that has been observed in obese individuals in previous studies (Compare et al., 2015). In contrast, a decreased Firmicutes/Bacteroidetes ratio has been observed in gut microbiota of non-obese patients with non-alcoholic fatty liver disease (NAFLD) (Wang et al., 2016). Similar to non-obese NAFLD, the Firmicutes/Bacteroidetes ratio decreased in ALD monkeys in our study, which suggests that Firmicutes/Bacteroidetes ratio may be a potential indicator for the risk of liver disease, and also indicates that the change of the F/B ratio may be connected to various diseases. In the present study, the abundance of Verrucomicrobia significantly increased in monkeys with ALD. In a previous report, the abundance of Verrucomicrobia was also observed as remarkably increased in high-fat diet fed mice (Xiao et al., 2017). In contrast, the abundance of Verrucomicrobiaceae significantly decreased in obese mice and non-alcoholic fatty liver cirrhotic patients (Kulecka et al., 2016; Ponziani et al., 2018). The results may indicate a difference between the intestinal microbiota of ALD and NAFLD.

In particular, we found that Lactobacillales and *Lactobacillus* significantly decreased in ALD monkeys compared with normal monkeys. Previous studies indicate that *Lactobacillus* can potentially affect the composition of intestinal microbiota and inhibit the expansion of harmful bacteria, protect the intestinal barrier through anti-inflammatory effects, therefore reducing liver pathologies (Ritze et al., 2014). In many clinical studies, *Lactobacillus* led to substantial reductions in the levels of ALT in 10 patients with non-alcoholic steatohepatitis (NASH) (Wong et al., 2013). *Lactobacillus* also can reduce features of NAFLD in humans (Li et al., 2010; Aller et al., 2011; Wong et al., 2013) and liver injury in mouse models (Li et al., 2010). In the present study, Lactobacillales and *Lactobacillus* significantly decreased in ALD monkeys. The reason may be that chronic alcohol administration reduced the capacity of the intestinal bacteria and indicates that *Lactobacillus* appears to have the potential for future therapies for ALD diseases. *Streptococcus* was found to be enriched in high-fat diet fed mice (He et al., 2018) and human cirrhotic patients with NAFLD (Ponziani et al., 2018). According to previous report, the development of obesity and atherosclerotic cardiovascular disease metabolic diseases are associated with these bacteria. *Streptococcus* enrichment in the stomach may be harmful (Karlsson et al., 2013; Jie et al., 2017; Korpela et al., 2017). However, the effect of alcohol intake on the abundance of streptococcus was not reported in human ALD patients or other animal models. In our case, the abundance of *streptococcus* tended to decrease in the monkeys of ALD, which indicates that the change of gut microbiota may be different between ALD, NAFLD, and obesity patients.

This intestinal microbiota plays a major role by helping to create the hosts' immune response and establishing the integrity of the gut mucosa (Xie et al., 2013). *In vitro* studies have shown that the effects on the growth of bacteria in the GI tract are directly and selectively caused by alcohol and overgrowth of this bacteria can produce ethanol which, in turn, can affect intestinal permeability (Baraona et al., 1986). Our study also demonstrates an important role of composition and diversity of the gut microbiota in the individuals with ALD and the compositional changes of gut microbiota by alcohol feeding might be linked to the progression of ALD. There is a clear separation at the gene level in the ALD and control groups, and the functional analysis of metagenomic data demonstrated that modulation of pathways involved in metabolism, biosynthesis, and enzyme families are associated with the ALD group. Energy metabolism decreased in monkeys with ALD. Our results are consistent to a previous study that showed energy metabolism is lower in patients with liver cirrhosis compared to healthy men (Qin et al., 2014).

Currently, there are no effective and universally accepted therapies for ALD (Diehl, 2002; Arteel, 2010). Chronic alcohol consumption can affect the composition and diversity of gut microbiota, which in turn affects liver function through gut microbiota changes and shifts. However, because social and environmental factors, including the place of residence (home, nursing home, or hospital), habitual diet, lifestyle, and medications taken are difficult to distinguish the effect of gut microbiota on the human body (Langille et al., 2014; Kim et al., 2016). Suitable animal models that accurately express the histological and metabolic features of human ALD will be of great benefit to the development of ALD drugs and therapies such as FMT. Control of the diet and environmental conditions can be homogeneous as well as easily maintained in non-human primate. Clinical progression of biochemistry, the composition of intestinal microbiota, and hepatic pathology are all presented accurately in this rhesus monkey model of alcohol-induced liver steatosis. Therapies including various diets, prebiotics, and probiotics might have the potential to impact and correct disturbances and act as potential future therapies for ALD. In addition, it has been reported that FMT is the most effective therapy for recurrence of *Clostridium difficile* infection (van Nood et al., 2013). FMT also has been proved to play an important role in therapy positive HBeAg in human (Ren et al., 2017). Base on these studies, it is speculate that FMT may modulate the severity of ALD according to the observation of the composition alteration of the intestinal microbiome in patients and ALD monkeys.

## Data Availability Statement

The raw data paired-ends reads were stored in the SRA online public database from NCBI with accession number SRP155721.

## Author Contributions

HW and YY conceived and designed the experiments, performed the experiments, wrote the paper, prepared figures and/or tables. XY did the statistical analyses and gut microbiome analysis. JW, YD, TH, and XL performed the experiments. LL and SL did histopathological analysis. BI edited the grammar and spelling. WJ and TT reviewed drafts of the paper. WS conceived and designed the experiments, contributed reagents, materials, and analysis tools, wrote the paper, reviewed drafts of the paper. All authors reviewed the manuscript.

### Conflict of Interest Statement

The authors declare that the research was conducted in the absence of any commercial or financial relationships that could be construed as a potential conflict of interest.

## Supplementary Material

Figure S1 The relative abundance of 8 orders showed significant difference between control and ALD monkeys.

Figure S2 The relative abundance of 30 genera showed significant difference between control and ALD monkeys.

Figure S3 The relative abundance of 50 species showed significant difference between control and ALD monkeys.
