# Supplementary figures and images for: Histopathological Features and Composition of Gut Microbiota in Rhesus Monkey of Alcoholic Liver Disease
Source: Front Microbiol. 2019 Feb 8;10:165. doi: 10.3389/fmicb.2019.00165 (PMC6375900; doi:10.3389/fmicb.2019.00165)

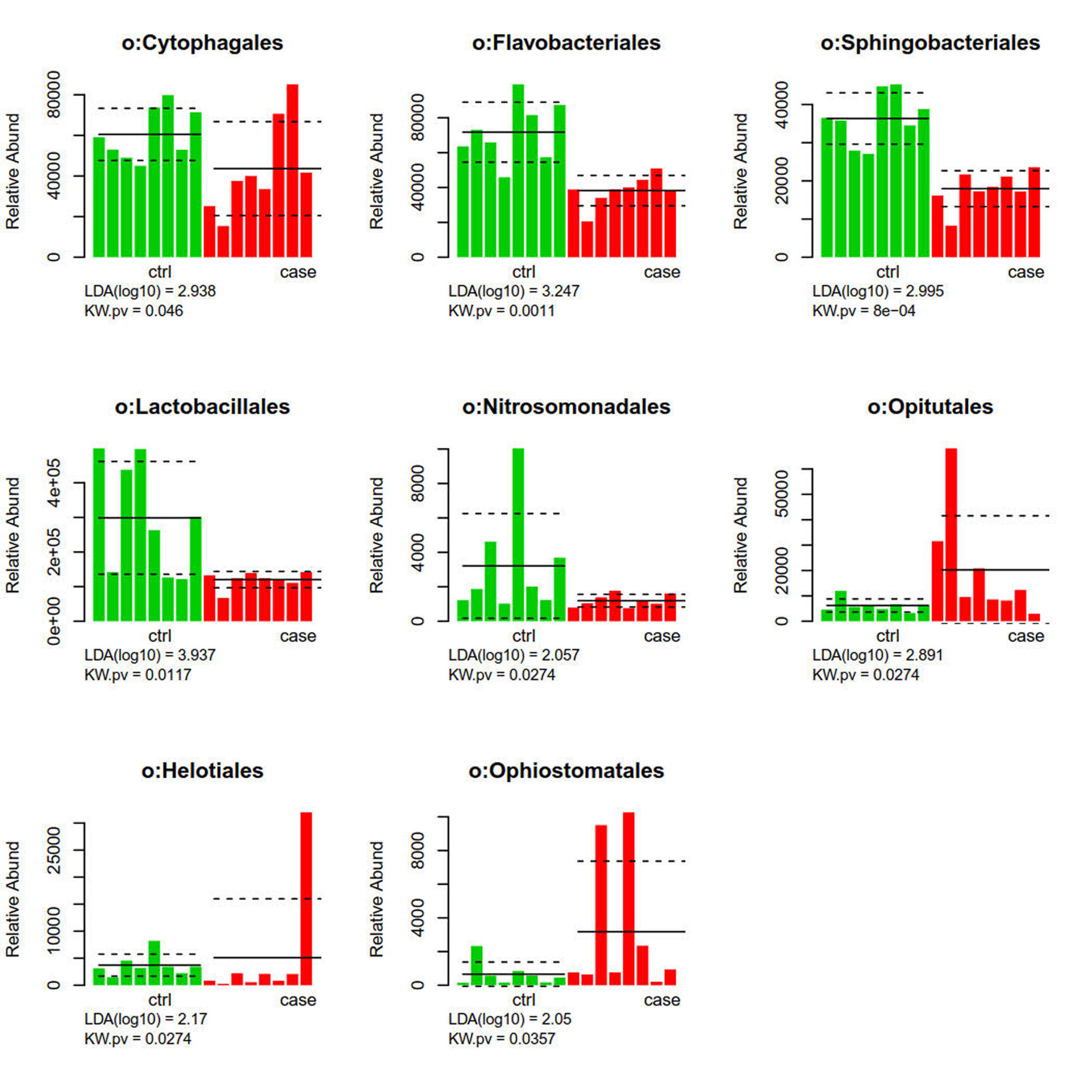

Supplement: Figure S1 — The relative abundance of 8 orders showed significant difference between control and ALD monkeys. [file Image_1.JPEG]

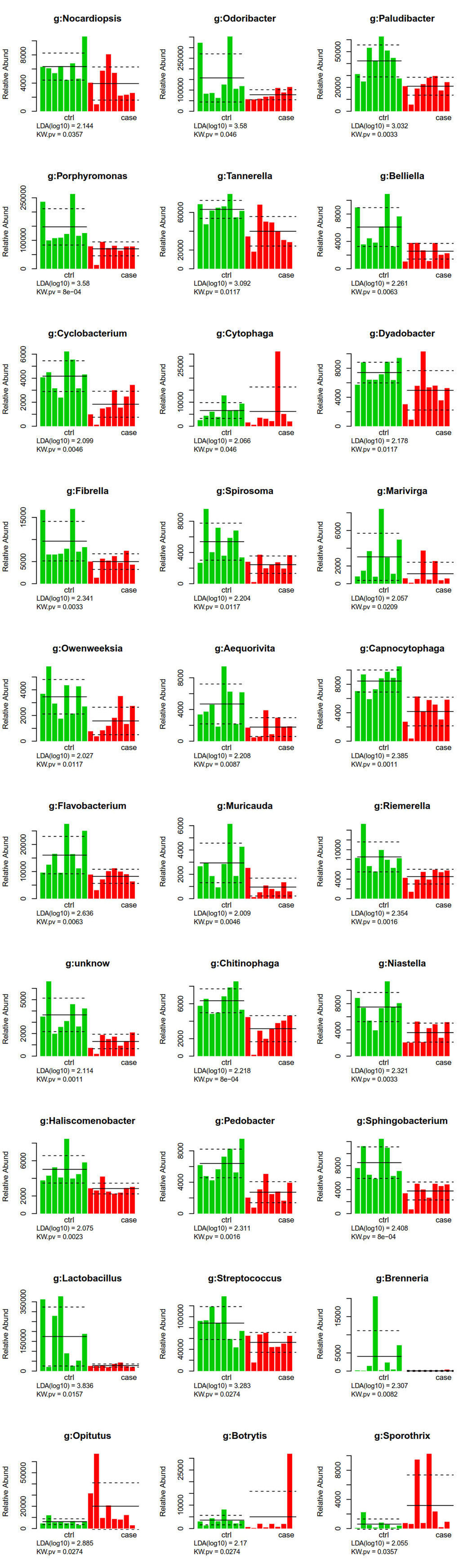

Supplement: Figure S2 — The relative abundance of 30 genera showed significant difference between control and ALD monkeys. [file Image_2.JPEG]

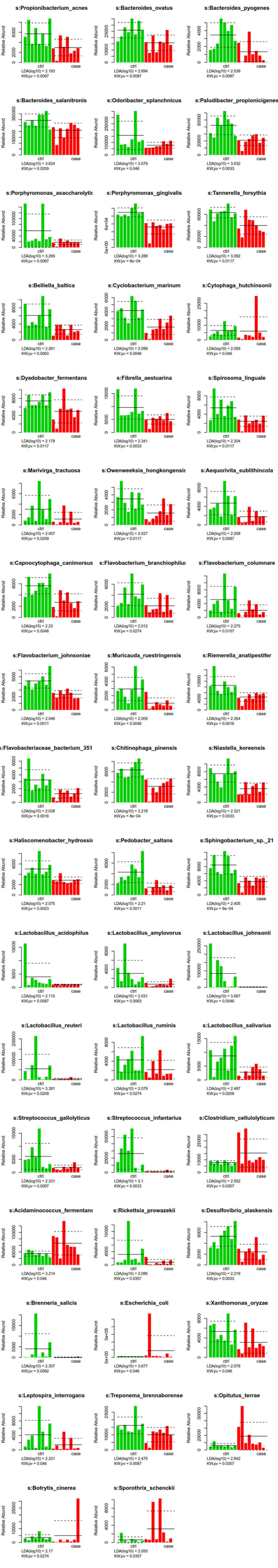

Supplement: Figure S3 — The relative abundance of 50 species showed significant difference between control and ALD monkeys. [file Image_3.JPEG]
